# Biodegradation of Zearalenone by a Novel *Bacillus* Strain X13 Isolated from Volcanic Rock Soil Using the Mycotoxin as the Sole Carbon Source

**DOI:** 10.3390/microorganisms13081954

**Published:** 2025-08-21

**Authors:** Di Meng, Kaizhong Xu, Jinbin Liu, Xiangru Liao

**Affiliations:** 1 Henan Provincial Engineering Research Center for Development and Application of Characteristic Microorganism Resources, Shangqiu Normal University, Shangqiu 476000, China; mengdi@sqnu.edu.cn; 2 Key Laboratory of Tropical Biological Resources of Ministry of Education, School of Life and Health Sciences, Hainan University, Haikou 570228, China; 3 School of Marine and Bioengineering, Yancheng Institute of Technology, Yancheng 224051, China; jinbin8810@ycit.edu.cn; 4 Key Laboratory of Industrial Biotechnology, Ministry of Education, Jiangnan University, Wuxi 214122, China

**Keywords:** mycotoxin degradation, volcanic soil isolate, carbon utilization, corn feed detoxification

## Abstract

Zearalenone (ZEN) is a widespread estrogenic mycotoxin that poses serious health risks to both humans and animals through the contamination of cereals and feeds. In this study, a novel *Bacillus* strain X13 was isolated from volcanic rock soil and demonstrated the unique ability to utilize ZEN as the sole carbon source for growth and metabolism. Under optimized conditions (37 °C, pH 8.0, and 5% inoculum in M9 minimal medium), strain X13 achieved a ZEN degradation efficiency of 98.57%. LC-MS analysis identified 1-(3,5-dihydroxyphenyl)-6′-hydroxy-1′-undecen-10′-one as the primary degradation product, indicating enzymatic hydrolysis of the lactone ring. Enzymatic assays revealed that the active components were extracellular, proteinaceous, and metal ion-dependent. Furthermore, the strain reduced ZEN content in mold-contaminated corn flour by 74.6%, effectively lowering toxin levels below regulatory limits. These findings suggest that *Bacillus* sp. X13 is a promising candidate for the bioremediation of ZEN-contaminated agricultural products, with significant potential for application in food and feed detoxification strategies. The robust degradation performance of strain X13 under simulated environmental conditions, combined with its adaptability to agricultural substrates, positions it as a viable solution for large-scale mycotoxin mitigation in the food industry chain, from pre-harvest field management to post-harvest storage processing.

## 1. Introduction

Zearalenone (ZEN) is a secondary metabolite produced by *Fusarium* constituting one of the most prevalent mycotoxins globally [1]. Structurally akin to estradiol, ZEN poses significant reproductive toxicity to both humans and mammals. Several studies have demonstrated its potential to induce infertility, abortion, and reproductive malformations in both human and animal populations [2]. Agricultural commodities and animal feeds frequently fall victim to ZEN contamination, with detectable levels commonly found in wheat bran, corn, and other agricultural products [3]. Prolonged exposure to ZEN could result in substantial economic losses within the realm of animal husbandry. Hence, addressing the issue of ZEN pollution in grains and feeds is imperative.

Currently, the effective strategy for addressing ZEN-contaminated feed is detoxification treatment. Reported ZEN detoxification methods encompass physical, chemical, and biological methods. Physical method primarily involves the utilization of materials for ZEN adsorption. For instance, Daković et al. reported the adsorption of ZEN by natural zeolitic tuff [4]. However, physical adsorption may inadvertently absorb nutrients in the feed, resulting in the diminishment of its nutritional value. Chemical methods, on the other hand, involve the use of ozone and other redox reagents to treat ZEN in feed [5,6]. Nonetheless, the application of chemical reagents may lead to pollution and the generation of toxic by-products. Moreover, traditional chemical and physical methods often exhibit unstable detoxification effects and can compromise feed quality. In contrast, biological methods offer notable advantages such as high efficiency, specificity, and mild reaction conditions. For instance, *Rhodococcus pyridinivorans* K408, as reported by Kriszt et al., demonstrated the ability to degrade 81.75% of ZEN post-cultivation [7]. Similarly, the fungus *Clonostachys rosea*, as reported by Kosawang et al., was found to degrade ZEN without generating harmful secondary metabolites [8]. However, three critical challenges hinder the industrial adoption of biological detoxification: (1) metabolic instability of degradative enzymes under fluctuating environmental conditions (e.g., temperature/pH variations during feed processing), (2) competition with indigenous microbiota in non-sterile agricultural substrates, and (3) economic constraints in biomass production for large-scale applications. Although numerous bacteria and fungi have been identified for their ZEN-degrading capabilities, the catalytic efficiency of reported strains often falls short, and reaction conditions remain relatively mild (typically 28–37 °C under neutral pH conditions). Particularly problematic is the rapid activity loss of most microbial detoxifiers when exposed to the acidic environments (pH 4.0–5.0) characteristic of silage and stored feeds. Thus, there is a need to screen for strains exhibiting higher catalytic efficiency and greater tolerance to acidic and high-temperature environments, while simultaneously addressing scalability through robust biomass production and formulation technologies.

In this study, strain X13, exhibiting high ZEN degradation activity, was screened from volcanic rock soil. Its species were identified through analysis of 16S rRNA and biochemical characteristics. The degradation characteristics of ZEN by strain X13 were investigated, along with the analysis of its degradation products and possible mechanisms. Concurrently, its efficacy in mitigating mold contamination in corn flour and its potential applications were explored.

## 2. Materials and Methods

### 2.1. Regents and Media

Reagents were obtained from the following suppliers: zearalenone (ZEN, Sigma-Aldrich, St. Louis, MO, USA); Ethylene Diamine Tetraacetic Acid (EDTA, Macklin, Shanghai, China); Sodium dodecyl sulfate (SDS) (Solarbio, Beijing, China); and proteinase K (Beyotime, Shanghai, China).

Volcanic rock soil was collected in Haikou City, Hainan Province (19°54′19″ N, 110°14′55″ E). Corn was purchased from Shangqiu’s primary agricultural marketplace. Soil samples were enriched in mineral salt medium (MSM) with ZEN. Subsequent strain cultivation was performed in five media types (Luria–Bertani broth (LB), Nutrient broth (NB), Tryptic soy broth (TSB), Minimal Medium M9, and brain heart infusion (BHI)), prepared either with or without ZEN supplementation as experimentally required. The compositions of culture media mentioned above are shown in Table S1. Unless otherwise specified, a final concentration of 10 μg/mL ZEN was mentioned throughout all experimental procedures in this study.

### 2.2. Detection of ZEN by LC-MS/MS

The ZEN was detected by a Liquid Chromatography–Tandem Mass Spectrometry (LC-MS/MS) system (QtrapTM 6500+, SCIEX, Framingham, MA, USA) equipped with a Kinetex^®^ C18 column (2.1 × 150 mm, particle 1.7 μm; Phenomenex, Torrance, CA, USA). Mobile phase A was water (0.1% formic acid and 5% acetonitrile), and mobile phase B was 95% acetonitrile (0.1% formic acid). The gradient program was set as follows: 0–5 min, increased from 25 to 70% B; 5–6 min, held at 70% B; 6–6.1 min, decreased from 70 to 25% B; 6.1–8 min, maintained at 25% B. The column temperature was maintained at 40 °C, with a flow rate of 0.3 mL/min and an injection volume of 5 μL. Eluents were determined in negative mode by electrospray ionization ion trap mass spectrometry. The nebulizer pressure was 30 psi. The drying gas flow rate was maintained at 6 L/min, with its temperature set to 350 °C. A collision energy of 24 eV was applied during analysis. The precursor ions and product ions of ZEN were 317.1 *m*/*z* and 175.0 *m*/*z*, respectively. The concentration of ZEN was determined according to the retention time of the standard compounds (ZEN) and the peak area of the ionic strength.

### 2.3. Screening and Isolation of Zearalenone Degrading Strains

A 2% (*w*/*v*) volcanic rock soil suspension was introduced into minimal salt medium (MSM) supplemented with ZEN and incubated for 24 h at 37 °C with constant shaking at 180 rpm. Following incubation, serial dilutions of the culture were prepared using physiological saline solution. Dilution solutions were coated on the ZEN-amended MSM solid medium and incubated at 37 °C for 24 h. The strains grown on the ZEN-amended MSM solid medium were selected and purified by streaking on a new LB plate. The preliminarily isolated strains were inoculated onto the fresh ZEN-amended LB medium and cultured at 37 °C with 180 rpm for 24 h. The ZEN-amended LB medium cultured under the same conditions was used as a control. The detection of ZEN content was described. The ZEN degradation rate was calculated according to (Equation (1)):

(1) $$\mathrm{ZEN}\;\mathrm{degradation}\;\mathrm{rate}\;(\%)=100\times \frac{\left(A_{0}-A_{1}\right)}{A_{0}},$$

where *A*_0_ is the peak area of the control group and *A*_1_ is the peak area of the experimental group.

### 2.4. Identification of Strain X13

Biochemical identification: The Gram staining of strain X13 was completed using a Gram staining kit (Biosharp, Hefei, China). The biochemical profile of strain X13 was determined using the HBI biochemical identification system (Hopebio, Qingdao, China). Citrate, propionate, D-xylose, d-arabinose, and D-Mannitol were used to detect the fermentation characteristics of strain X13. The Voges–Proskauer (V-P) test was employed according to the methods of Barritt [9]. Gelatin liquefaction capability, 7% NaCl tolerance, growth at pH 5.7, nitrate reduction capacity, and starch hydrolysis activity were tested. The V-P test was used to detect whether strain X13 can ferment glucose to produce acetylmethyl methanol.

For phylogenetic analysis, genomic DNA was isolated from strain X13 using the TIANamp Bacterial DNA Extraction Kit (TIANGEN, Beijing, China) following the manufacturer’s protocol. The complete genome obtained served as the basis for subsequent PCR. The 16S rRNA gene was amplified from the whole genome of strain X13 using 16S primers (27F: 5′-AGAGTTTGATCCTGGCTCAG-3′; 1492R: 5′-TACGGCTACCTTGTTACGACTT-3′). 16S rRNA gene sequencing of strain X13 was performed by Tsingke Biotech (Beijing, China), followed by homologous alignment using the NCBI Blast Tool [10]. Multiple sequence alignment of the 16S rRNA gene was performed through ClustalW 2 [11]. The genetic phylogenetic tree of strain X13 was constructed by MEGA 11 software using the Neighbor-Joining algorithm [12].

### 2.5. Effect of Culture Conditions on ZEN Degradation by Strain X13

#### 2.5.1. Effect of Culture Medium on ZEN Degradation by Strain X13

Strain X13 cultured overnight at 37 °C was diluted to 0.5 McFarland standard with sterile saline. The dilutions of strain X13 were aseptically transferred (1% *v*/*v* inoculum) into fresh ZEN-supplemented media, including LB, NB, TSB, BHI, and M9. Cultures were incubated at 37 °C with orbital shaking (180 rpm) for 24 h. Different ZEN-amended media (LB, NB, TSB, BHI, and M9 media) were used as controls. The ZEN concentration was detected, and the degradation capacity was calculated. Unless otherwise specified, all subsequent inoculations used standardized strain X13 cultures adjusted to the 0.5 McFarland standard.

#### 2.5.2. Effect of Culture Time on ZEN Degradation by Strain X13

Strain X13 was inoculated (1% *v*/*v*) into the ZEN-amended M9 medium and cultured at 37 °C, 180 rpm. ZEN concentrations and cell density (OD_600_) were monitored at 4–48 h intervals, with the uninoculated medium as a control.

#### 2.5.3. Effect of Inoculum Size on ZEN Degradation by Strain X13

Strain X13 was inoculated at varying volumes (1–5% *v*/*v*) into the ZEN-amended M9 medium and cultured at 37 °C, 180 rpm for 24 h. ZEN concentrations and cell density (OD_600_) were monitored, with the uninoculated medium as a control.

#### 2.5.4. Effect of Initial pH on ZEN Degradation by Strain X13

Strain X13 (5% inoculum) was cultured in the ZEN-amended M9 medium at varying pH (3–9), 37 °C, and 180 rpm for 24 h. ZEN concentrations and cell density (OD_600_) were monitored, with the uninoculated medium as a control.

#### 2.5.5. Effect of Temperature on ZEN Degradation by Strain X13

Strain X13 (5% inoculum) was cultured in the ZEN-amended M9 medium at various temperatures (20–42 °C) with shaking (180 rpm) for 24 h. ZEN concentrations and cell density (OD_600_) were monitored, with the uninoculated medium as a control.

#### 2.5.6. Effect of ZEN Concentration on ZEN Degradation by Strain X13

Strain X13 (5% inoculum) was cultured in the M9 medium containing varying ZEN concentrations (1.25–40 μg/mL) at 37 °C with shaking (180 rpm) for 24 h. ZEN concentrations and cell density (OD_600_) were measured against corresponding ZEN-containing controls.

### 2.6. Localization of ZEN-Degrading Active Substances in Strain X13

Strain X13 (5% inoculum) was cultured in the ZEN-amended M9 medium (37 °C, 180 rpm, 24 h). The cultured bacterial fluid was centrifuged at 8000× *g* and 4 °C for 15 min. After centrifugation, cells and fermentation supernatants were collected separately. The cell suspension was subjected to three sequential washing steps with phosphate-buffered saline (PBS; 20 mM, pH 7.2), using equivalent volumes for each wash cycle. Following resuspension, cells were lysed via ultrasonication (600 W, 15 min). The resulting lysate was centrifuged (8000× *g*, 15 min, 4 °C) to separate the soluble fraction (supernatant) from cellular debris. For preparation of heat-inactivated controls, cell suspensions were incubated at 100 °C for 15 min prior to analysis. The relative ZEN degradation activities of different components (bacterial fluid, cells, heat-inactivated cells, fermentation supernatants, and cell disruption supernatant) were detected separately. The reaction system was carried out in a temperature-controlled shaking incubator maintained at 37 °C with constant agitation (180 rpm) for 12 h. The control of the bacterial fluid group and the fermentation supernatants group was the ZEN-amended M9 medium. The PBS (pH 7.2, 20 mM) with ZEN was used as the control for the other three groups (cell group, cell disruption supernatant group, and heat-inactivated cell group). The concentration of ZEN in the systems was 10 µg/mL.

To further verify whether ZEN degradation was mediated by enzymes in cell-free fermentation supernatant, the supernatant was treated with proteinase K, heating, SDS, and EDTA. The experimental procedure was as follows:

The cell-free fermentation supernatant was treated with proteinase K at a final concentration of 200 μg/mL, followed by incubation at 37 °C for 1 h to ensure complete protein digestion. The fermentation supernatant without proteinase K was used as a control. The heat treatment of the fermentation supernatant was carried out by heating at 100 °C for 15 min. The fermentation supernatant without heating treatment was used as a control. The fermentation supernatant was added to SDS (10%, *w*/*v*) and incubated at 37 °C for 30 min. A parallel control was maintained without SDS addition. Separate aliquots of supernatant were amended with EDTA (5 mM final concentration) and similarly incubated at 37 °C for 30 min. Corresponding EDTA-free controls were processed identically. All treatment groups and controls were spiked with ZEN to a final concentration of 10 μg/mL. The reaction mixtures were then incubated with constant agitation (180 rpm) at 37 °C for 12 h to assess degradation efficiency. The ZEN content in the system was detected by LC-MS.

### 2.7. Analysis of ZEN Degradation Products by Strain X13

Strain X13-mediated ZEN degradation was performed under the previously determined optimal conditions. The resulting metabolic products were characterized using liquid chromatography–mass spectrometry with ion trap-time of flight detection (LCMS-IT-TOF; Shimadzu, Tokyo, Japan). Separation was achieved on an ACQUITY UPLC BEH C18 column (Waters, Milford, MA, USA) following the chromatographic conditions described in reference [13]. Mobile phase A was water (0.1% formic acid and 5% acetonitrile). Mobile phase B was 95% acetonitrile (0.1% formic acid). The gradient was set as 0–1 min, 10% B; 1–7 min, 10–100% B; 7–9 min, 100% B; 9–9.01 min, 100–10% B; 9.01–10 min, 10% B. The system flow rate was 0.3 mL/min. The injection volume was 3 µL. Eluents were determined in positive mode by electrospray ionization ion trap mass spectrometry. Full-scan mode detection was used with a scan range from *m*/*z* 100 to 400.

### 2.8. Detoxification of Moldy Maize by Strain X13

The detoxification of moldy corn flour by strain X13 was performed according to the method reported previously, with the appropriate modifications [14]. Briefly, contaminated corn kernels were mechanically pulverized using an industrial grinder. One gram of corn flour (containing 1.81 μg ZEN) was mixed with 3.5 mL of PBS (pH 7.2, 20 mM) and 0.5 mL of a logarithmic-phase culture of strain X13 (OD_600_ = 1.0 ± 0.1), while control samples received a heat-inactivated bacterial suspension (100 °C, 15 min). The reaction mixtures were incubated in 15 mL polypropylene tubes at 37 °C with orbital shaking (180 rpm) for 12 h in the dark. Following incubation, samples were dried at 60 °C for 8 h, then extracted with 4 mL of 80% (*v*/*v*) acetonitrile and centrifuged (8000× *g*, 15 min, 4 °C) to collect supernatants. The supernatants were evaporated under nitrogen gas and reconstituted in 1 mL of 50% (*v*/*v*) acetonitrile, followed by filtration through 0.22 μm nylon membranes. The content of ZEN in the resuspended samples was detected using LCMS-IT-TOF (Shimadzu, Tokyo, Japan). The heat-inactivated strain X13 solution was used as a control.

### 2.9. Statistical Analysis

All experiments were carried out at least in triplicate. Statistical analysis was performed GraphPad Prism 9 (GraphPad Software, San Diego, CA, USA). Parametric tests were applied as all datasets met the assumptions of normal distribution (assessed by Shapiro–Wilk tests) and homogeneity of variance (confirmed by the Brown–Forsythe test). One-way analysis of variance (ANOVA) coupled with post hoc Tukey’s test was used for multiple group comparisons. Two-tailed paired *t*-tests were employed for pairwise comparisons. All values are presented as the mean ± standard deviation (SD), with statistical significance set at *p* < 0.05. Data visualization was performed using the same software.

## 3. Results and Discussion

### 3.1. Screening and Isolation of Zearalenone Degrading Strains

Volcanic rock soil is rich in trace elements and metal elements, which makes it a fertile reservoir of microbial resources [15]. In this study, 20 potential ZEN-degrading strains were isolated, utilizing ZEN as the sole carbon source. Among these strains, strain X13 exhibited the most promising degradation efficacy towards ZEN (Figure S1). The retention time of ZEN in the LC-MS/MS system was determined to be 5.8 min (Figure S2a). The ion intensity peak area of ZEN degraded by strain X13 decreased significantly at 5.8 min. Furthermore, a new peak emerged at a retention time of 3.62 min (Figure S2b), suggesting the degradation of ZEN by strain X13 and the formation of new metabolites. Following 24 h cultivation in the LB medium, strain X13 achieved a ZEN degradation rate of 70.66%. Given its superior efficacy in ZEN degradation, subsequent investigations prioritized the study of strain X13.

### 3.2. Identification of Strain X13

**Figure 1:**
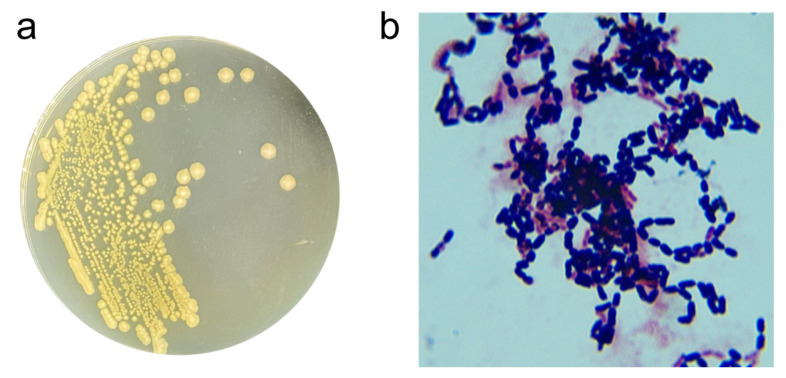
Morphological traits of strain X13. Colony morphology of strain X13 (**a**); Gram-staining of strain X13 (magnification 1000×) (**b**).

The ZEN-degrading strain X13 was isolated and purified on LB solid plates following the guidelines outlined in Bergey’s Manual Systematic Bacteriology [16]. Colonies of strain X13 cultivated on LB plates exhibited a flat, round morphology. Initially, the colony color was white, transitioning to light yellow upon growth (Figure 1a). Microscopic examination revealed strain X13 to be Gram-positive and short rod-shaped (Figure 1b). Further physiological and biochemical traits of strain X13 were assessed to ascertain its taxonomic classification (Table S2). A positive V-P test indicated that strain X13 had the ability to utilize glucose for acetylmethyl carbinol production. Strain X13 demonstrated the ability to utilize D-arabinose, D-mannitol, and D-glucose, while showing no utilization of citrate, propionate, or D-xylose. Further physiological characterization revealed that strain X13 was incapable of nitrate reduction. However, the strain demonstrated notable environmental adaptability, showing robust growth at pH 5.7 and maintaining viability in media containing up to 7% (*w*/*v*) NaCl. Additionally, strain X13 exhibited gelatin liquefaction and starch hydrolysis abilities. Notably, strain X13 was capable of utilizing ZEN as the sole carbon source for growth (Figure S3). The physiological and biochemical traits of strain X13 were consistent with those reported for *Bacillus* species [17].

**Figure 2:**
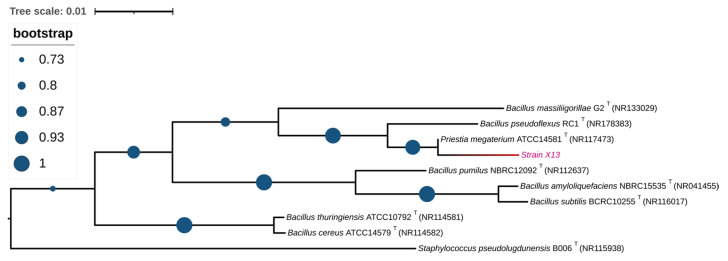
Phylogenetic analysis of strain X13 based on 16S rRNA. Type strains are labeled with a “T”. The branch containing strain X13 is shown in red.

In order to identify the strain at the molecular level, the 16S rRNA sequence of strain X13 was amplified via PCR and subsequently deposited into NCBI with the GenBank ID PP837864. Upon comparison in the NCBI database, the 16S rRNA sequence of strain X13 exhibited over 99% similarity to that of *Priestia megaterium* (*Bacillus megaterium* prior to 2020). Furthermore, a phylogenetic tree based on 16S rRNA sequences of various *Bacillus* species revealed that strain X13 shared the closest genetic relationship with *Priestia megaterium* (Figure 2). Therefore, strain X13 was classified as a member of the *Bacillaceae* family. *Bacillus* species are recognized as safe probiotics characterized by rapid reproduction and robust stress resistance [18]. Previous studies have reported the ZEN degradation capabilities of *Bacillus* strains such as *B. subtilis*, *B. amyloliquefaciens*, and *B. pumilus* [19,20].

### 3.3. Effect of Culture Conditions on ZEN Degradation by Strain X13

**Figure 3:**
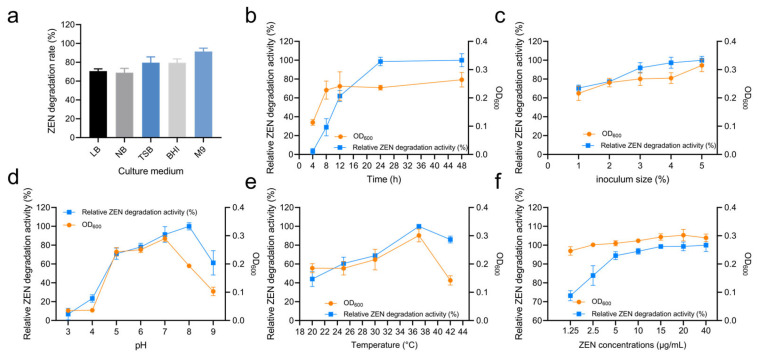
Degradation characteristics of ZEN by strain X13. Effect of culture medium on strain X13 growth and ZEN degradation (**a**). Effect of culture time on ZEN degradation by strain X13 (**b**). Effect of inoculum size on ZEN degradation by strain X13 (**c**). Effect of initial pH on ZEN degradation by strain X13 (**d**). Effect of temperature on ZEN degradation by strain X13 (**e**). Effect of ZEN concentration on ZEN degradation by strain X13 (**f**).

Different culture media can provide varying nutrient elements such as carbon sources, nitrogen sources, and growth factors. In this study, the impact of different media on the ZEN degradation by strain X13 was investigated. As summarized in Table S3 and Figure 3a, strain X13 exhibited the highest ZEN degradation rate (91.46%) in the M9 medium, with lower rates observed in LB (70.66%), NB (68.99%), TSB (79.60%), and BHI (80.28%). These findings suggest that strain X13 demonstrates a superior degradation effect in relatively nutrient-deficient media compared to nutrient-rich media. It is postulated that this phenomenon may be attributed to the ability of strain X13 to utilize ZEN as the sole carbon source (Figure S3). Currently, most reported ZEN-degrading strains have been evaluated in nutrient-rich media. For example, the reported *B. amyloliquefaciens* D-1 can effectively degrade ZEN in an LB medium [21]. However, strain X13, described in this study, displayed ZEN degradation capabilities in the M9 medium, which is relatively nutrient-deficient. This characteristic is advantageous for reducing fermentation costs and facilitating the development of biological agents.

Figure 3b and Table S3 summarize the effect of cultivation time on ZEN degradation. Within the first 12 h, the optical density (OD) of strain X13 cells rapidly increased, corresponding with an escalation in ZEN degradation activity. Specifically, after 12 h of cultivation, the growth of strain X13 entered a stationary phase, while relative ZEN degradation activity continued to rise rapidly. Although the highest degradation activity was observed after 48 h of cultivation, there was only marginal improvement in ZEN degradation activity compared to the 24 h mark. Similar studies also reported that the degradation rate of *B. amyloliquefaciens* D-1 stabilizes at a high value (80%) after 24 h of ZEN degradation [21]. Thus, a cultivation time of 24 h was considered optimal for subsequent experiments.

The effect of different inoculum sizes on the ZEN degradation by strain X13 was investigated using the M9 medium and at 37 °C. The optimal inoculum size for ZEN degradation was found to be 5%, with no significant difference in bacterial growth or ZEN degradation beyond this value after 24 h (Figure 3c and Table S3). This could be due to the limited nutritional content of the medium, as higher inoculum volumes may have minimal effect on microbial activity. Previous studies have indicated that inoculum size can impact the metabolic conditions and stress responses of microorganisms [22]. Additionally, higher inoculum volumes may facilitate the germination of *Bacillus* spores, thereby enhancing the response to external environmental changes [23].

The pH value plays a pivotal role in microbial growth and enzymatic reactions [24]. The effect of pH on ZEN degradation by strain X13 was evaluated across a broad range (pH 5–9), as shown in Figure 3d and Table S3. Strain X13 exhibited robust growth and maintained high ZEN degradation activity with optimal degradation at pH 8. Even at pH 9, the degradation activity remained above 60%, highlighting the strain’s tolerance to alkaline conditions. Given that alkaline reagents are commonly employed in feed processing [25], probiotics capable of thriving in alkaline environments hold a competitive edge. Similar studies have also reported the effective ZEN degradation capabilities of *B. licheniformis* CK1 across a pH range of 2.5–8 [26].

Temperature also significantly affected ZEN degradation by strain X13. As shown in Figure 3e and Table S3, degradation activity increased with temperature (20–37 °C), reaching its maximum at 37 °C. Elevated temperatures generally promote microbial growth and enzymatic reaction rates. However, excessively high temperatures can induce biological structural instability and enzyme inactivation [27]. In this study, strain X13 maintained over 50% ZEN degradation activity across a temperature range of 25–42 °C, demonstrating broad temperature adaptability. Similarly, the optimal ZEN degradation temperature for the reported *Clostridium sporogenes* F39 was found to be 35 °C [28]. Additionally, a novel microbial consortium (NZDC-6) was reported to withstand temperatures of up to 60 °C while degrading ZEN [29].

Strain X13 also exhibited concentration-dependent ZEN degradation, as summarized in Figure 3f and Table S3. The degradation activity increased rapidly at low concentrations (1.25–15 µg/mL), then plateaued at higher concentrations (15–40 µg/mL). Notably, ZEN concentrations up to 40 µg/mL did not inhibit growth and even stimulated biomass production at moderate levels (1.25–20 µg/mL), potentially contributing to the enhanced degradation activity. These results demonstrate remarkable tolerance of strain X13 to ZEN across a wide concentration range (1.25–40 µg/mL). Similarly, the reported *B. subtilis* Y-33 exhibited tolerance to 20 µg/mL of ZEN [30]. The rate of ZEN degradation reaction was contingent upon the initial ZEN concentration. For instance, the reported ZEN degradation rates of *B. natto* CICC24640 at 0.02 mg/mL and 5 mg/mL were 100% and 18%, respectively [31].

### 3.4. Localization of ZEN-Degrading Active Substances in Strain X13

**Figure 4:**
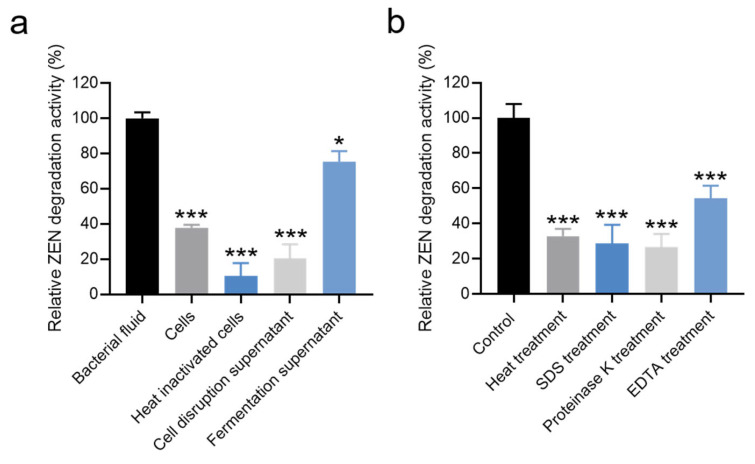
Effects of different components (**a**) and treatment conditions (**b**) of strain X13 on ZEN degradation. Statistical significance among groups was determined by Tukey’s post hoc test. * *p* < 0.05, *** *p* < 0.001.

The relative ZEN degradation activities of different components (bacterial fluid, cells, heat-inactivated cells, fermentation supernatants, and cell disruption supernatant) were assessed individually. As depicted in Figure 4a and Table S4, the bacterial fluid exhibited the highest relative ZEN degradation activity. The relative ZEN degradation activity of the fermentation supernatant was 75.33%. In contrast, the relative ZEN degradation activities of cells and cell disruption supernatant were 37.89% and 20.57%, respectively. Notably, the relative ZEN degradation activity of heat-inactivated bacteria was only 10.48% (Figure 4a and Table S4). These results suggest that the ZEN degradation activity of strain X13 predominantly originates from extracellular supernatant and intracellular substances. During fermentation, *Bacillus* typically secretes various enzymes. It is presumed that the enzymes in the supernatant play a crucial role in ZEN degradation. Similarly, Liu and colleagues reported that the primary ZEN degradation active components of *B. spizizenii* B73 were derived from extracellular enzymes in the fermentation supernatant [32].

To determine whether extracellular enzymes were responsible for ZEN degradation, cell-free fermentation supernatant was subjected to various treatments. As shown in Figure 4b and Table S5, treatments significantly affected the ZEN degradation capacity of the fermentation supernatant. Compared to the untreated control (100% activity), the relative degradation activities were reduced to 32.93% after heat treatment (100 °C, 15 min), 28.81% following SDS (10% *w*/*v*) exposure, and 26.84% after proteinase K (200 μg/mL) digestion. Notably, EDTA (5 mM) treatment caused the most substantial inhibition, decreasing degradation activity by 45.51% relative to the control (Figure 4b). Heating can induce alterations in the spatial structure of proteins [33], while SDS treatment is known to disrupt the secondary structure of proteins [34]. Additionally, proteinase K can cleave peptide bonds linked to the carboxyl terminus of hydrophobic, sulfur-containing, and aromatic amino acids [35]. EDTA, functioning as a metal ion chelating agent, can sequester metal ions in solutions and proteins [36]. These results provide compelling evidence supporting our hypothesis that proteinaceous enzymes in the fermentation supernatant are primarily responsible for ZEN degradation. The significant reduction in activity following proteinase K treatment (26.84% residual activity) and heat inactivation (32.93% residual activity) strongly implicates heat-labile proteins as the key catalytic components. Furthermore, the pronounced 45.51% decrease in degradation activity after EDTA chelation suggests that certain metalloenzymes, requiring essential metal cofactors, contribute substantially to the ZEN degradation capacity of strain X13.

### 3.5. Analysis of ZEN Degradation Products by Strain X13

**Figure 5:**
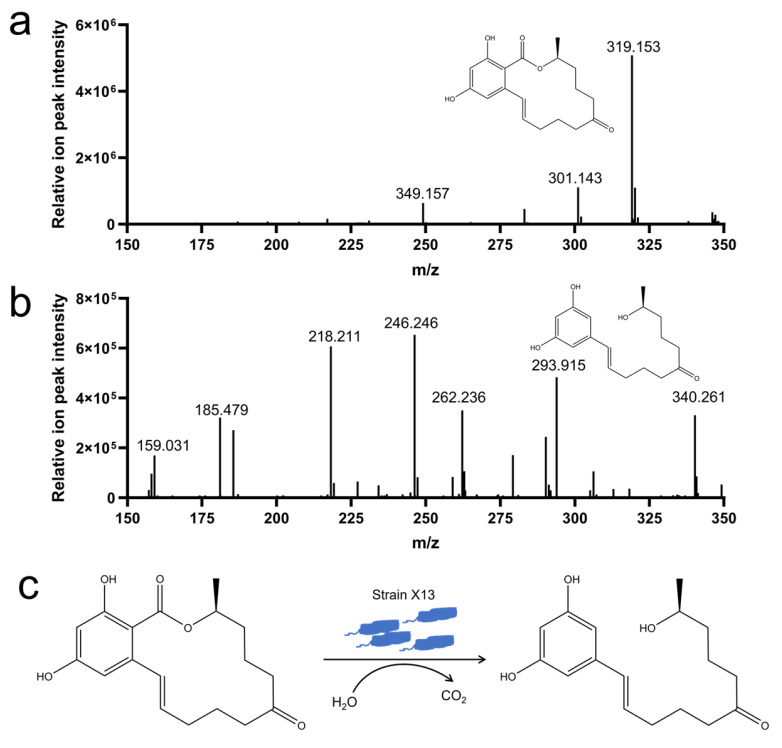
Analysis of ZEN degradation products by strain X13. ZEN (*m*/*z* 319) (**a**); 1-(3,5-dihydroxyphenyl)-6′-hydroxy-1′-undecen-10′-one (*m*/*z* 293) (**b**); proposed mechanism for ZEN degradation by strain X13 (**c**).

To elucidate the degradation mechanism of ZEN by strain X13, LC-MS analysis was employed to examine the degradation products of ZEN. In the control samples, characteristic peaks exhibited a mass of 319 *m*/*z*, corresponding to the mass of ZEN (Figure 5a). Notably, the ZEN degradation product detected at 293 *m*/*z* was consistent with the mass of 1-(3,5-dihydroxyphenyl)-6′-hydroxy-1′-undecen-10′-one (Figure 5b and Table S6). Consequently, it is presumed that under the catalysis of strain X13, ZEN undergoes hydrolysis to form 1-(3,5-dihydroxyphenyl)-6′-hydroxy-1′-undecen-10′-one (Figure 5c). The characteristic macrolide structure of zearalenone (ZEN), particularly its lactone moiety, suggests that enzymatic hydrolysis of the ester bond represents a probable degradation mechanism. This ring-opening transformation would generate corresponding linear carboxylic acid derivatives, which could be subsequently metabolized through additional enzymatic steps. Previous reports have indicated that the lactonohydrolase ZHD101, derived from *C. rosea*, can cleave ZEN to produce 1-(3,5-dihydroxyphenyl)-6′-hydroxy-1′-undecen-10′-one, which exhibits reduced toxicity [37]. The reported biodegradation mechanisms of ZEN encompass hydroxy ketone reduction, double-bond cleavage, hydroxylation, methylation, glycosylation, and ring opening [38]. It is plausible that strain X13, as reported in this study, may employ multiple mechanisms for ZEN degradation simultaneously, necessitating further investigation.

### 3.6. Detoxification of Moldy Corn by Strain X13

**Figure 6:**
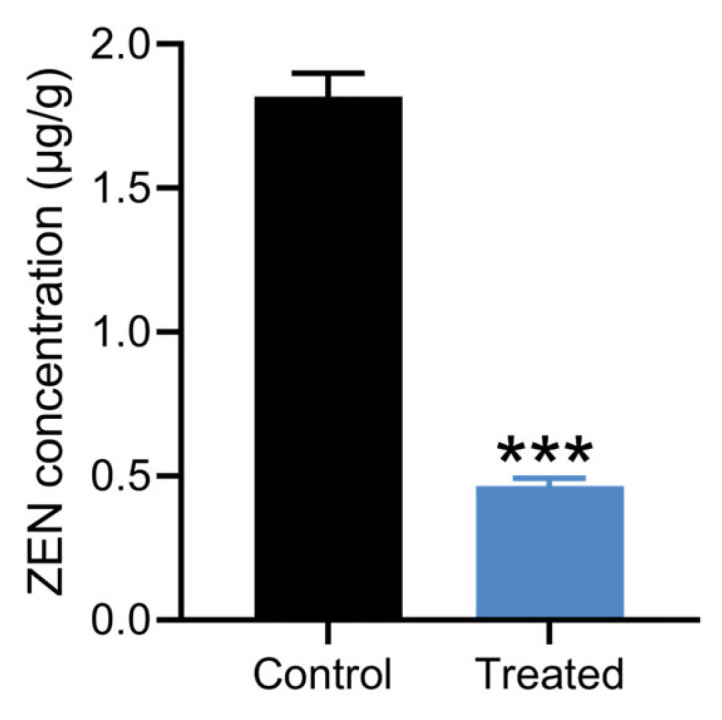
Detoxification of moldy corn by strain X13. The treated group was corn treated with strain X13. The control group was corn treated with heat-inactivated strain X13. *** indicates extremely significant differences between control group and treated group (*p* < 0.001) based on Tukey’s test.

Corn, grain, and silage are among the most common foods contaminated by ZEN [2]. Strain X13 was employed to detoxify moldy corn. The LC-MS spectrum of the ZEN standard exhibits a prominent peak at a retention time of 7.1 min (Figure S4a). Following treatment with strain X13, the peak area of moldy corn at the retention time of 7.1 min was markedly diminished (Figure S4b,c). As depicted in Figure 6 and Table S7, in comparison with the control group, the ZEN content in corn flour of the strain X13 treatment group decreased by 74.6%. This result surpasses the efficacy reported for *Aspergillus niger* FS10, which removed 60.01% of ZEN from corn soaking [39]. Following biological treatment with strain X13, the ZEN concentration in contaminated corn flour was significantly reduced from 1.81 μg/g to 0.46 μg/g (*p* < 0.001, n = 3) (Figure 6 and Table S7). The final ZEN concentration (0.46 μg/g) not only fell below the European Commission’s regulatory threshold for compound feed for calves, dairy cattle, sheep, and goats (0.5 μg/g) [40], but also demonstrated the remarkable detoxification efficiency of strain X13. These results highlight the strong potential of strain X13 as a biocontrol agent for ZEN contamination in agricultural commodities, particularly for post-harvest remediation of mycotoxins in feed ingredients.

While strain X13 shows high detoxification efficiency, further validation is needed to assess (1) the safety and toxicity of ZEN degradation products in vivo, (2) the stability and activity of the enzyme(s) under industrial feed processing conditions (e.g., high temperature, prolonged storage), and (3) scalability in pilot- or industrial-scale trials. Addressing these gaps will be critical for practical application in the feed industry.

## 4. Conclusions

In this study, strain X13 with high ZEN degradation activity was successfully screened from volcanic rock soil. Strain X13 was identified as *Bacillus* sp. by 16S rRNA sequencing. After optimization of degradation conditions, the degradation rate of ZEN by strain X13 under the optimal conditions (37 °C, pH 8.0, inoculum 5%, and M9 medium) was 98.57%. The degradation of ZEN by strain X13 mainly originated from its extracellular enzyme secreted into the fermentation supernatant. The degradation products of ZEN were analyzed by LC-MS, and it was presumed that ZEN was hydrolyzed to 1-(3, 5-dihydroxyphenyl)-6′-hydroxy-1′-undecen-10′-one under the catalysis of strain X13. Furthermore, utilizing corn as the medium, it was observed that strain X13 effectively removed 74.6% of ZEN from moldy corn flour. These findings indicate promising prospects for the application of strain X13 in ZEN degradation and detoxification of corn feed. For future industrial applications, scale-up fermentation processes could be optimized in bioreactors, and strain X13 may be developed as a probiotic feed additive through lyophilization or microencapsulation to enhance stability. Further research should focus on enzyme purification, structural characterization, and immobilization techniques to improve catalytic efficiency and reusability for large-scale feed detoxification.

## Data Availability

The data presented in this study are openly available in GenBank ID: PP837864. [GenBank] [https://www.ncbi.nlm.nih.gov/nuccore/PP837864.1/] [PP837864] (accessed on 31 May 2024).

## Supplementary Materials

The following supporting information can be downloaded at: https://www.mdpi.com/article/10.3390/microorganisms13081954/s1, Figure S1: Degradation of ZEN by strains isolated from volcanic soil; Figure S2: LC-MS/MS ion flow diagram of Zen degraded by strain X13; Figure S3: Growth of strain X13 in a culture medium with ZEN as the sole carbon source; Figure S4: LC-MS ion flow diagram of moldy corn; Table S1: Culture medium used in this study; Table S2: Biochemical and physiological of strain X13; Table S3: Degradation characteristics of ZEN by strain X13; Table S4: Effects of different components of strain X13 on ZEN degradation; Table S5: Effects of different treatment conditions of strain X13 on ZEN degradation; Table S6: Identification of the ZEN degradation products by strain X13; Table S7: ZEN reduction in moldy corn flour by strain X13 vs. EU regulatory limits.
