# Supplementary material for: Biodegradation of Zearalenone by a Novel Bacillus Strain X13 Isolated from Volcanic Rock Soil Using the Mycotoxin as the Sole Carbon Source
Source: Microorganisms. 2025 Aug 21;13(8):1954. doi: 10.3390/microorganisms13081954 (PMC12388560; doi:10.3390/microorganisms13081954)
Supplement: Supplementary file 1 [file microorganisms-13-01954-s001.zip › microorganisms-3802153-supplementary.pdf]

Supplementary materials

Table S1. Culture medium used in this study.

Medium type	Component	Concentration (g/L)
Mineral salt medium (MSM)	Na_2HPO_4	6.8
	KH_2PO_4	3
	NaCl	0.5
	NH_4Cl	1
	$MgSO_4$	0.24
	$CaCl_2$	0.011
Luria-Bertani broth (LB) medium	yeast extract	5
	peptone	10
	NaCl	10
Nutrient broth (NB) medium	beef extract powder	3
	peptone	10
	NaCl	5
Tryptic soy broth (TSB) medium	tryptone	17
	soybean papain hydrolysate	3
	glucose	2.5
	K_2HPO_4	2.5
	NaCl	5
M9 medium	Na_2HPO_4	6.8
	KH_2PO_4	3
	NaCl	0.5
	NH_4Cl	1
	glucose	4
	$MgSO_4$	0.24
	$CaCl_2$	0.011
Brain heart infusion (BHI) medium	peptone	10
	dehydrated calf brain soaked powder	12.5
	dehydrated beef heart soaked powder	5
	NaCl	5
	glucose	2
	Na_2HPO_4	2.5

Table S2. Biochemical and physiological of strain X13.

Biochemical reaction	Strain X13 a
V-P test	+
Citrate	-
Propionate	-
D- xylose	-
D-arabinose	+
D-mannitol	+
Gelatin liquefaction	+
7% NaCl	+
pH 5.7	+
Nitrate reduction	-
Starch hydrolysis	+
Zen utilization	+

[a] "+" indicates positive reaction, "-" indicates negative reaction.

Table S3. Degradation characteristics of ZEN by strain X13.

Parameter	Tested range	Relative ZEN degradation activity (%)	OD_{600}
Medium	LB	77.26±2.417	ND
	NB	75.44±4.545	ND
	TSB	87.03±6.102	ND
	BHI	87.04±4.021	ND
	M9	100.00±3.593	ND
Time (h)	4	3.33±3.036	0.11±0.010
	8	29.00±9.110	0.23±0.032
	12	62.04±5.907	0.24±0.051
	24	98.62±4.529	0.24±0.008
	48	100.00±6.951	0.26±0.026
Inoculum size (%)	1	70.54±3.095	0.22±0.025
	2	77.36±1.273	0.25±0.015
	3	91.99±5.560	0.27±0.023
	4	97.3±5.860	0.27±0.019
	5	100±4.118	0.32±0.022
pH	3	6.82±6.122	0.04±0.001
	4	23.49±3.929	0.04±0.002
	5	70.99±5.940	0.24±0.015
	6	78.12±3.963	0.25±0.011
	7	91.40±8.321	0.29±0.010
	8	100.00±3.842	0.19±0.006
	9	61.22±12.960	0.10±0.015
Temperature (°C)	20	44.16±8.004	0.19±0.016
	25	60.65±6.600	0.19±0.024
	30	68.91±2.390	0.22±0.036
	37	100.00±1.399	0.30±0.023
	42	86.02±3.600	0.14±0.016
ZEN concentrations (μg/mL)	1.25	73.22±2.663	0.25±0.015
	2.5	83.93±5.232	0.27±0.008
	5	94.48±2.103	0.27±0.009
	10	96.76±1.511	0.28±0.004
	15	99.31±0.701	0.30±0.012
	20	99.38±2.312	0.30±0.020
	40	100.00±3.305	0.29±0.014

"ND" indicates not determined.

Table S4. Effects of different components of strain X13 on ZEN degradation.

Component	Relative ZEN degradation activity (%)
Bacterial fluid	100.00±3.470
Cells	37.90±1.543
Heat inactivated cells	10.48±7.230
Cell disruption supernatant	20.57±7.818
Fermentation supernatant	75.33±5.981

Table S5. Effects of different treatment conditions of strain X13 on ZEN degradation.

Treatment Condition	Relative ZEN degradation activity (%)
Control (untreated)	100.00±7.940
Heat treatment	32.94±4.083
SDS treatment	28.81±10.520
Proteinase K treatment	26.84±7.279
EDTA treatment	54.49±7.105

Table S6. Identification of the ZEN degradation products by strain X13.

Retention time (min)	Measured mass [M + H]+ (m/z)	Common name	Proposed structure	References
4.5	293	1-(3, 5-dihydroxyphenyl)-6’-hydroxy-1’-undecen-10’-one	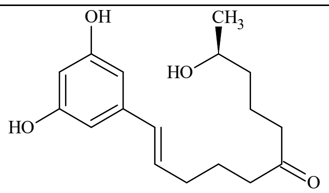	[1, 2]

[1] Cai, P.; Liu, S.; Tu, Y.; Shan, T. Toxicity, biodegradation, and nutritional intervention mechanism of zearalenone. *Sci Total Environ* **2024**, *911*, 168648, doi:10.1016/j.scitotenv.2023.168648.

[2] Liu, X.; Wu, N.; Zhang, M.; Xue, F.; Xu, Q. Isolation and characterization of the zearalenone-degrading strain, *Bacillus spizizenii* B73, inspired by esterase activity. *Toxins (Basel)* **2023**, *15*, 488, doi:10.3390/toxins15080488.

Table S7. ZEN reduction in moldy corn flour by strain X13 vs. EU regulatory limits.

Parameter	Value
Initial ZEN concentration	1.81 μg/g
Final ZEN concentration	0.46 μg/g
% Reduction	74.6%
EU Limit (Compound Feed)	0.5 μg/g, Commission Recommendation (EU) 2016/1319 for calves, dairy cattle, sheep, goats.

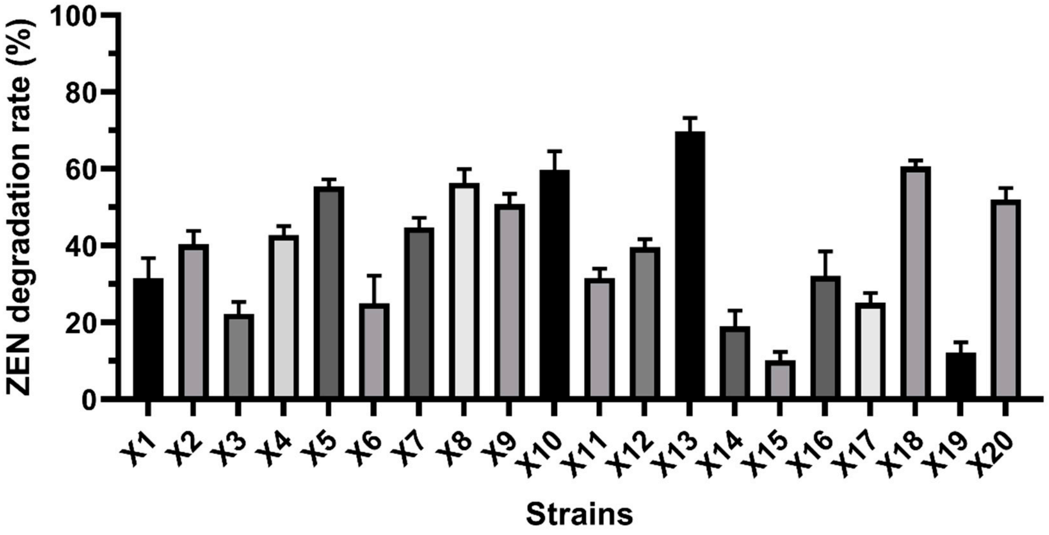

Figure S1. Degradation of ZEN by strains isolated from volcanic soil. All strains were inoculated into LB medium containing ZEN (10 μg/mL) and cultured at 37°C and 180 rpm for 24 h. LB medium containing ZEN (10 μg/mL) was used as control.

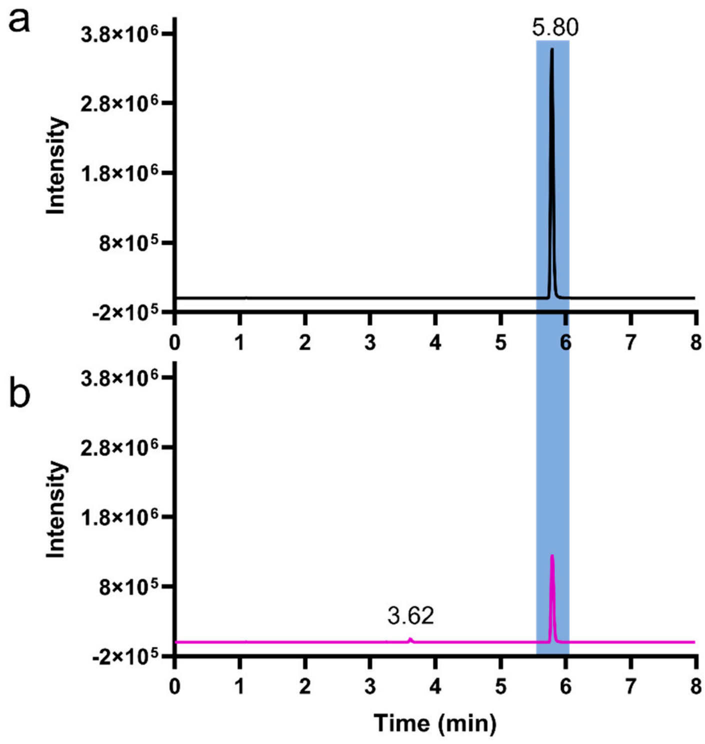

Figure S2. LC-MS/MS ion flow diagram of Zen degraded by strain X13. ZEN Control group (a); Zen degraded by strain X1 (b). The mobile phase A was water (0.1% formic acid and 5% acetonitrile) and the mobile phase B was 95% acetonitrile (0.1% formic acid). The gradient was set as: 0-5 min, 25-70% B; 5-6 min, 70% B; 6-6.1 min, 70-25%B; 6.1-8 min, 25%B. The precursor ions and product ions of Zen were 317.1 *m/z* and 175.0 *m/z*, respectively.

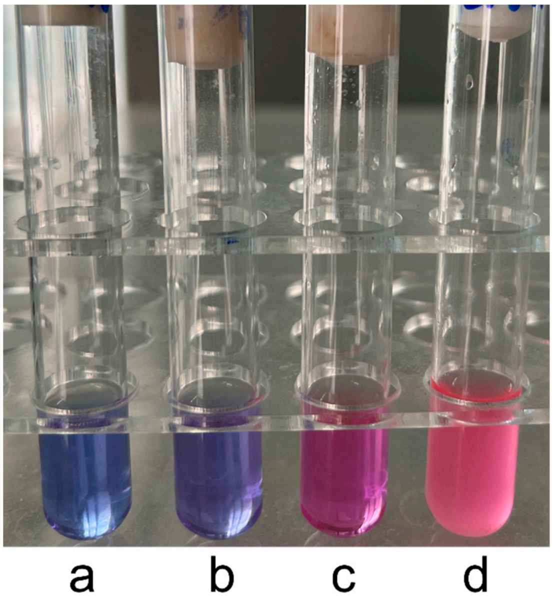

Figure S3. Growth of strain X13 in a culture medium with ZEN as the sole carbon source. MSM medium without carbon source (a); MSM medium containing ZEN (5 μg/mL) (b); MSM

medium containing ZEN (10 μg/mL) (c); MSM medium containing glucose (4 mg/mL) (d). The single colony of Strain X13 was taken into sterile water to adjust the concentration to 0.5 McFarland standard, and inoculated into four liquid media (a, b, c, d) with 0.1% (v:v) inoculation amount. After cultivation at 37 °C and 180 rpm for 48 h, resazurin (final concentration 10 μg/mL) was into the media. Observe color changes after standing for 30 min. The darker the red color, the more bacterial cells there are.

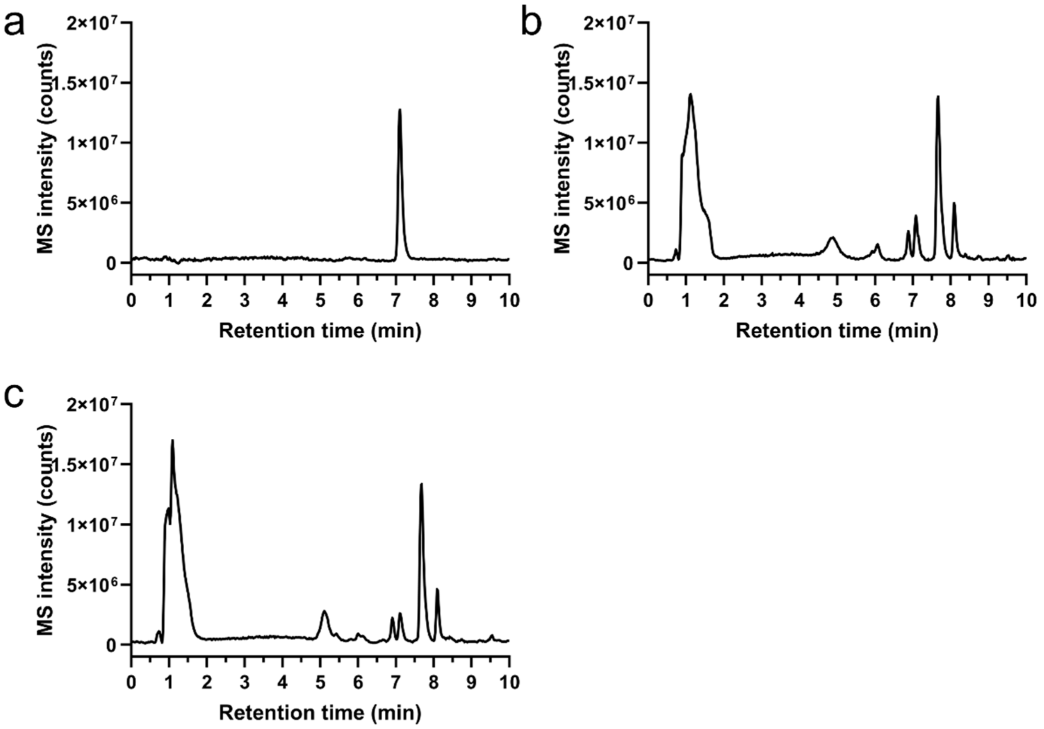

Figure S4. LC-MS ion flow diagram of moldy corn. ZEN (a); moldy corn treated with Heat inactivated strain X13 (b); Moldy corn after treatment with strain X13 (b).
